# Affording unavoidable emergency surgical care – The lived experiences and payment coping strategies of households in Ibadan metropolis, Southwestern Nigeria

**DOI:** 10.1371/journal.pone.0232882

**Published:** 2020-05-20

**Authors:** Taiwo Obembe, Sharon Fonn

**Affiliations:** 1 School of Public Health, University of Witwatersrand, Johannesburg, South Africa; 2 Department of Health Policy and Management, University of Ibadan, Ibadan, Nigeria; University Complutense of Madrid, SPAIN

## Abstract

**Background:**

Pre-payment and risk pooling schemes, central to the idea of universal health coverage, should protect households from catastrophic health expenditure and impoverishment; particularly when emergency care is required. Inadequate financial protection consequent on surgical emergencies occurs despite the existence of risk-pooling schemes. This study documented the experiences and coping strategies of slum and non-slum dwellers in a southwestern metropolis of Nigeria who had undergone emergency surgery.

**Methods:**

In-depth interviews were conducted with 31 participants (13 slums dwellers, 18 non-slum dwellers) who had recently paid for emergency surgical care in Ibadan. Patients who had experienced catastrophic health expenditure from the use of emergency surgical care were identified and people who paid for the care were purposively selected for the interviews. Using an in-depth interview guide, information on the experiences and overall coping strategies during and after the hospitalization was collected. Data were analyzed inductively using the thematic approach.

**Results:**

The mean age of the 31 participants (consisting of 7 men and 24 women) was 31 ± 5.6years. Apathy to savings limited the preparation for unplanned healthcare needs. Choice of hospital was determined by word of mouth, perceptions of good quality or prompt care and availability of staff. Social networks were relied on widely as a coping mechanism before and during the admission. Patients that were unable to pay experienced poor and humiliating treatment (in severe cases, incarceration). Inability to afford care was exacerbated by double billing and extraneous charges. It was opined that health care should be more affordable for all and that the current National Health Insurance Scheme, that was operating sub-optimally, should be strengthened appropriately for all to benefit.

**Conclusion:**

The study highlights households’ poor attitude to health-related savings and pre-payment into a social solidarity fund to cover the costs of emergency surgical care. It also highlights the factors influencing costs of emergency surgical care and the role of social networks in mitigating the high costs of care. Improving financial protection from emergency surgical care would entail promoting a positive attitude to health-related savings, social solidarity and extending the benefits of social health insurance.

## Background

Emergency care is an essential component of health systems. Needed sudden care may be medical [1] or surgical [2] and is often critically urgent to avoid aggravated morbidity or mortality. Emergency surgery is defined as ‘*surgical procedures that cannot be delayed*, *for which there is no alternative therapy*, *and for which a delay could result in death or permanent impairment of health*’ [2]. Globally, emergency surgical procedures totaled over 230 million cases about a decade ago [2] and emergency cesarean section (CS), comprises a significant proportion of the 18.5 million CS cases worldwide [3]. Sub-Saharan Africa has the highest proportion of disability-adjusted life years (DALYs) due to surgical conditions estimated at 38 DALYs per 1,000 population [4]. The high burden of emergency care in low- and middle income countries (LMIC) has been attributed to factors such as lack of adequate health care resources, unorganized systems [5], that are complicated by the migration of health care professionals [6] and cost factors [7–9]. Despite evidence of the cost-effectiveness of surgery [10], many LMIC households are still unable to afford it.

The readiness and ability to afford care for sudden health challenges have been variably evaluated across many populations [11,12]. Ability of households to afford the cost of health care is important because affordability issues can totally truncate access [13] and depends primarily on household income [14]. Delays in care are related to financial costs [15,16], that is usually borne out of lack of preparedness[14]. Other factors affecting choice of a service range from home initiated interventions to patronage of drug shops for convenience[17], knowledge and perceived severity of the ailment [18], cleanliness and structure (aesthetics) of hospitals and availability of specialists or proficient staff. Also, health seeking behaviours influence the accessibility and affordability to emergency care. Care-seeking factors include delays arising from ignorance, cost of care and lack of adequate means of transportation [19,20]. The resulting delay has important implications for outcome of surgical procedures [21]. When health care is not affordable, patients resort to extreme coping strategies that range from selling off household assets to borrowing, which leads to further impoverishment [22–26].

Universal Health Coverage (UHC) defined, by World Health Organization (WHO), as “access to key promotive, preventive, curative, and rehabilitative health interventions for all at an affordable cost, thereby achieving equity in access”[27], is a notable intervention to address readiness of health care end-users and improve affordability of emergency surgical care. UHC schemes, including social insurance schemes [28,29], health savings account initiative [14], and free healthcare programmes supported by citizen participation and realistic and informed budgets [30,31], are initiatives that have been used to improve financial access to emergency care. Financing UHC, either through taxation or contributory insurance scheme, involves revenue collection, pooling of resources, and purchasing services. Where the tax base cannot sustain health financing, UHC has advocated pre-payment schemes to help reduce impoverishment, consequent on catastrophic health expenditure[27,32]. Such pre-paid schemes rely on social solidarity (where premiums or subsidies are exerted on all, irrespective of health/wealth status) to make health care accessible and affordable when needed [33].

Despite global call to embrace universal health coverage, UHC service coverage index in Nigeria is estimated at 39% [34]. Nigeria’s premier UHC scheme, the National Health Insurance Scheme (NHIS) was officially signed into law in 1999 but not fully operational until 2005. The scheme consists of an obligatory system to enroll people in formal employment, a voluntary system and a system to cover designated vulnerable groups [35]. Total enrolment, nonetheless, has been sub-optimal as only about 5 million people (approximately 3% of the population) are covered by the scheme today [36]. Since its inception, the formal sector social health insurance programme (FSSHIP) that covers organised private sector and federal government employees [36], is the most functional program under the scheme [37]. Enrolment is mandatory for all formal sector employees based on a 5% monthly contribution from every worker’s salary and 10% of the worker’s basic salary paid by the employer. Coverage extends to enrollee’s spouse and four dependents under 18years of age [38].

About 70 percent of Nigerians live on less than $1 a day with approximately 35% of the 70 percent living in absolute poverty while over 90 percent live on less than $2 a day [39,40]. In Nigeria, just as in other regions of the world [41], the aspiration for a better life has also fueled urbanization promoting people to increasingly seek employment in urban areas. However, insufficient investment in infrastructure [42] combined with unplanned migration promotes proliferation of slums that are typically characterized by poor living conditions, absent infrastructure and poor access to health care [43]. As of 2014, 50.2% of Nigeria’s urban population were living in slums [44]. The slum dwellers (poor and vulnerable groups) constitute a substantial proportion of the urban informal sector in Nigeria [32] and face considerable hardship when needing health care which may range from physical access to affordability issues.

Another characteristic of the Nigerian health care system is dependence on out-of-pocket payment as a major funding mechanism [45]. The resultant financial burden on families (particularly with poorer families) has been linked to varying difficulties in access to care and susceptibility to catastrophic expenditures, especially when paying out-of-pocket [46]. There has been some research on households’ experiences and payment coping strategies for emergency health services in Nigeria [47,48]. This study set out to explore the lived experiences of people that were admitted for a recent emergency surgical procedure in selected hospitals of a southwestern metropolis of Nigeria with a specific focus on both slum and non-slum dwellers.

## Methods

### Study setting

Ibadan, the capital city of Oyo State, is a prominent transit point between the coastal region and the areas to the north [49]. Its population is estimated to be 3,720,643 people [50] and it is the third most populous city in Nigeria. Ibadan has five metropolitan local government areas and eight urban-rural local government areas [51] inhabited predominantly by the Yorubas. The metropolis is home to slums that are interspersed across the metropolitan local government areas. These core slums areas are usually heterogeneous ethnically and professionally [52]. Other slums areas found in Ibadan are smaller and are located on the periphery of the city [52].

### Study design, sampling and participants

This is a qualitative descriptive study that is nested in a larger quantitative study. The 5 local government areas in Ibadan metropolis were selected purposively to capture the core slums within the city core. Out of a total of 197 secondary facilities and one tertiary facility (University College Hospital) situated across the region, 16 facilities were selected (consisting of six public facilities, nine private secondary facilities and the only tertiary facility in the metropolis) using simple random sampling. Index cases–people who required emergency care—were identified in these sites. To be eligible, surgical emergencies had to be a general surgical or an emergency caesarian section (from obstetric wards). Emergency surgical needs in children (below 18 years), neurological or orthopedic surgeries (requiring extended hospital stays of over one month period) were not eligible.

People who were identified as being responsible for paying the costs of the hospitalization were enrolled as participants in the study–this could have also been the person who was hospitalized. Those found to have incurred catastrophic health expenditure (CHE), in a larger study, were invited to participate in this qualitative study as the study cases. CHE, which is defined as spending on healthcare exceeding a certain income threshold [53], was set at 5% of the participant’s annual income for the present hospitalization.

All the eligible participants were placed on an ordered list. Participants were contacted from the first payer on the list; these included payers that had confirmed willingness to be interviewed and had provided written consent at the recruitment phase. If a payer declined to participate or was not available to be interviewed, the next payer on the ordered list was contacted. Participants were recruited and enrolled one by one and the process was continued until the 31^st^ interview when data saturation occurred. Thus, a total of 31 participants were interviewed.

### Data collection

In-depth interviews were conducted with the aid of interview guides conducted in both English and Yoruba. The guide was developed based on the study objectives to capture in-hospital experiences, post-operative views and coping strategies guided by relevant literature. The interview guide was translated into Yoruba by a native Yoruba speaker who is also an English language expert and translated back to English. This was carried out to ensure that the constructs, which questions aimed to explore, were not lost in both English and Yoruba versions of the interview guide. The interview guide was tested on selected participants that had recently undergone emergency surgery in hospital facilities outside the study setting in order to achieve face validation. All interviews were carried out by two persons–the facilitator (who moderated the sessions) and a research assistant (that acted as both the note taker and time keeper). Face to face interviews were conducted in locations and at times suggested by the participants in order to accommodate their personal schedules. Fifteen of the interviews were conducted in local language (Yoruba) while sixteen interviews were conducted in English. Information on recent hospitalization and experiences related to payment for health care was obtained using funneling and probing as the main interview strategies [54,55]. Credibility of data was ensured through respondent validation (member check). Respondent validation was carried out by obtaining feedback from the participants concerning the accuracy of the data they had supplied and also the researcher’s interpretation of the data [56].

### Data analysis

All interviews were transcribed verbatim and then translated into English if required. The transcribed data were imported into Atlas.Ti vs 7.5.21 [57]. Interview texts were coded into themes using an inductive approach [58]. In this approach, transcripts were read through and familiarization was established. Codes were subsequently assigned to emergent themes and subthemes to explain the social phenomenon [59]. Three analysts reviewed the data and then agreed on what codes and themes had emerged from the data. One of the researchers was an independent researcher who did not participate in the interviews. Themes were constructed so that there were no overlaps and so that the themes were robust enough to capture the data. The demographic characteristics that describe the participants are reported as counts and percentages.

### Ethical consideration

Approval to conduct this study was obtained from the Human Ethics Review Committee of the University of Witwatersrand (M170284), University of Ibadan /University College Hospital Ethical committee (UI/EC/17/0006) and Oyo State Ministry of Health (AD13/479/123). Written, informed consent was obtained from every participant for participation, audio-recording of interviews and note taking. To ensure confidentiality, anonymous identifiers were used and access to data restricted to only those directly involved in the study.

## Results

The findings of this study are presented according to four major themes namely preparing for unplanned healthcare needs, pre-surgical experience, post-surgical experience, and strategies to improve affordability. Whereas two sub-themes emerged from the pre-surgical experience–choosing a service and getting a service; post-surgical experience comprised three sub-themes including payment for service, helping with paying for service, and feelings about service. Strategies to improve affordability are summarized under two main themes: federal level policy or program reform and individual/social level intervention. While the study was set up to compare experiences between slum and non-slum dwellers, we did not find differences between these two groups and we have not presented the data by dwelling status. Table 1 shows the socio-demographic characteristics of participants.

**Table 1:** Socio-demographic characteristics of the participants.

		Slum		Non-Slum	
		Frequency (n)	Percent (%)	Frequency (n)	Percent (%)
**1**	**Age-group (N = 31)**				
	**0–25 years**	2	15.4	3	16.7
	**26-35years**	9	69.2	13	72.2
	**>36 years**	2	15.4	2	11.1
**2**	**Gender (N = 31)**				
	**Male**	4	30.8	3	16.7
	**Female**	9	69.2	15	83.3
**3**	**Religion (N = 31)**				
	**Christianity**	7	53.8	17	94.4
	**Islam**	6	46.2	1	5.6
**4**	**Educational Status (N = 31)**				
	**Primary**	5	38.5	1	5.6
	**Secondary**	5	38.5	2	11.1
	**Tertiary**	3	23.1	12	66.7
	**Post-grad**	0	0.0	3	16.7
**5**	**Marital Status (N = 31)**				
	**Single**	2	15.4	5	27.8
	**Married**	11	94.6	12	66.7
	**Separated**	0	0.0	1	5.6
**6**	**Ethnicity (N = 31)**				
	**Yoruba**	13	100	18	100
**7**	**Type of Hospital (N = 31)**				
	**Private**	10	76.9	3	16.7
	**Public**	3	23.1	15	83.3
**8**	**Occupation (N = 31)**				
	**Formal**	1	7.7	11	61.1
	**Informal**	12	92.3	6	33.3
	**Unemployed**	0	0.0	1	5.6
**9**	**Grade of Hospital (N = 31)**				
	**Secondary**	11	84.6	3	16.7
	**Tertiary**	2	15.4	15	83.3

### Sociodemographic characteristics of participants

Thirteen of the participants were slum dwellers and 18 were non-slum dwellers. The participants ranged in age from 21–46 years with a mean age of 31 ± 5.6 years. (Table 1). Majority of the participants were aged 26–35 years for slum (69.2%) and non-slum dwellers (72.2%). Males constituted a minority among the slum dwellers (30.8%) and even fewer among the non-slum dwellers (16.7%). Christianity was the predominant religion practiced by participants who did not live in slums (94.4%), while a mix of Islamic and Christian worshippers was reported among the slum dwellers. More of the non-slum dwelling payers (66.7%) attained a minimum of tertiary education while primary and secondary education was the highest level of education attained among the slum dwelling payers. Non-slum payers were predominantly formally employed (61.1%) unlike the slum dwellers where the majority were informally employed (92.3%) (Table 1)

### Preparing for unplanned health care needs

Majority of the participants had no money, savings or health insurance to fall back on during the admission; saving for care was anathema to them. It was found that apathy to savings limited the preparation for unplanned healthcare needs or anticipation for a hospital admission with phrases such as, “***who would want to save to pay a doctor”*?** A 31year-old slum dweller opined:

*…Well*, *the person involved in savings would have in mind what he intends or wants his savings for*. *And he would have also made plans for how he will invest the proceeds of the contribution into his business to make gains*. *No one would want to save just for the purpose of spending it in the hospital you know*…

- ***R1_F_SD_31years_private***

Furthermore, it was also perceived that one could be inviting a health challenge (medical ailment) for oneself when saving for such.

### Choosing a service

The choice of a hospital was largely determined by the structure, organisation of hospital services and human resources for many of the participants. This varied from hospital to hospital. Broadly speaking, the choice of a hospital was dependent on aesthetics, perceptions about human resources available and type of facility (private or public). On probing for the choice of facility with respect to human resources, choices with regards to skilled staff were determined by the reputation of the facility, which was gleaned by word of mouth.

*“…*..*I asked for the hospital that was good and it was recommended to me that it is a good place to give birth and for medical attention…*…”

***R9_F_SD_28years_private***

Many of the participants were satisfied with the service delivery received in private facilities compared to public facilities. The expertise of the service rendered with respect to timeliness, competence and professionalism was rated excellent. A 31-year-old slum dweller that sought care in a private facility opined:

*“When it comes to care here*, *they are excellent*. *And when we talk about timeliness*, *they are also good*. *Their competence and professionalism are also very good*. *I was rest-assured with all these qualities…*.*”* ***R1_F_SD_31years_private***

Of interest is that while the cost of services was expected to be a determinant of how people chose services, this did not come up during the interviews despite a probing question to explore this. This was however an important feature of post admission experiences.

### Experience of the service

Emerging themes with regards to aesthetics revealed that patients who were admitted in private facilities were satisfied with the cleanliness of the environment while many participants that sought treatment in the public facilities were not satisfied.

*“Inside and outside*, *toilet and bathroom and everything*, *the cleaners are cleaning and working every minute*. *After some have finished and have gone*, *another set of people will resume and come forth*. *The place is neat”…*..***R17_F_SD_46years_private***

*“That one na error (laughs) like the first time I came in*, *there were no bed sheets; I used my wrapper (cover cloth) for a long time before they changed it*. *When you even ask for a bedsheet*, *it would seem as if they are doing you a favour*. *They said that laundry section did not have bed sheets*. *Maybe they were not dried or something”*.

***…*..*R12_F_NSD_28years_public***

Once admitted, participants experienced variations in hospital payment procedures and options with initiation of care. Some private hospitals operated the ***“treat first-pay later***” policy. However, for the hospitals that operated this “*treat first-pay later*” policy, patients had to provide a guarantor (prominent family member in the patient’s social network) that was required to sign an undertaking to pay full complete costs later on in the admission or before discharge. Other hospitals (public facilities) allowed patients to pay piecemeal subject to the availability of funds. Both private and public facilities provided some initial care (first aid) if a down payment of the total estimated bill could not be paid upfront. However, treatment would be stopped after the patient had been stabilized and would not resume until payment was completed.

*“…*.*I told them to please help me that they should do everything that they need to do for the mother and the baby to be alive that I will pay but the doctor on duty then said that they won’t do anything if I don’t bring the money”*. ***R17_F_SD_46years_private***

### Payment for the service and consequences for inability to pay

Both slum and non-slum dwellers experienced some difficulty paying hospital bills that had to be paid for during their hospital admission. Participants (particularly at public facilities) reported double billing while those using private facilities were billed for what they had been asked to buy out of pocket before the surgery, such as consumables. Some, at private facilities, were charged for items that they considered to be the responsibility of the facility to provide. For example one participant reported having to pay for the fuel needed for the facility’s generator.

*“They said at the time they wanted to do the operation that there was no light so the money for petrol to put in the generator equals 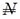
35*,*000”****—R17_F_SD_46years_private***

Post-surgery, many facilities employed a ‘*detention policy’* which meant that if no payment was received, patients were not discharged. Though commoner among the private facilities, some public facilities also operated a detention policy. For many of the participants, this was neither acceptable nor convenient.

*“…Yes*, *I was delayed because of money problem so I was a bit delayed”*. ***R3_F_NSD_42years_public***

*“…Like if I get 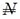 5000*, *I will pay it*, *even there was a time I was owing their pharmacy 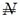
3*,*000+ but I had to pay all before allowed to go—****R16_M_SD_27yrs_public***

The participants attested to unfavorable experiences while being kept in the hospitals until payment was completed in full. Dehumanizing practices and maltreatment were reported in some hospitals. Some payers described feeling humiliated when they were unable to pay or failed to obtain needed support from social networks. There were cases where patients had been incarcerated in the hospital for a prolonged period of time.

*“I noticed there that when you don’t pay there is syringe they pass through you*, *they passed almost two on my hand I had to be shouting that they should come and remove one that is already aching me but they refused that if I didn’t pay*, *they wouldn’t remove the syringe…*.. ***R9_F_SD_28years_private***

Some hospitals had systems in place to support selected households that experienced difficulty paying hospital bills. These ranged from total waivers to partial waivers (discounts). This approach was more common in the tertiary hospital and was only granted to patients based on the severity of the surgical emergency and the age of the patient. Children and pregnant women were more likely to be granted opportunities of total or partial waivers if they could convince the hospital management of a genuine inability to pay.

### Help with paying for services

The option that payers used most when they could not pay was to rely on social networks for financial assistance. Social networks and the need to maintain good social relations with those networks was considered to be important. Maintaining excellent interpersonal relations was considered crucial to securing and receiving financial assistance from families and friends when in dire financial emergencies. For instance, worthiness to be lent money was a validation that the borrower was a person with integrity (who can be trusted) and that he/she can pay back easily after the surgery. Many normalized the act of borrowing from stable social networks (family and friends) when in financial crisis. Nonetheless, it was also the case that some families and networks could fail when turned to for financial assistance, irrespective of excellent inter-personal relationships as illustrated by the report:

*“it helps a lot if you have been good to the family and if the families like to help*, *but you know some family will not help you since they want you dead; they will say “****too ba le ku koku****”(if you want to die*, *please go ahead*).***—R16_M_SD_27yrs_public***

In instances, when close social networks including families and friends disappoint, wider and more distal social networks (such as those related to religious affiliations/bodies), are explored. According to a 42-year old, non-slum participant, her immediate family did not assist her in any way during her admission. However, some members of her religious affiliation provided support:

*…all my family ran away because of expenses but thank God I go to church so people in my church really helped me*.***—R3_F_NSD_42yrs_public***

### Feelings about the service (post-surgical experience)

When we explored how interviewees coped with the payment issues, they initially said they felt fine about it. There was a tendency to minimize the burden and also to place it out of their control as an inevitable event. In order to feel comfortable with these catastrophic consequences, they normalized their experiences and behaviours to justify that they had or were coping well. For many, it was only on further probing that it was observed that their experiences were not as manageable as they initially suggested. Responses typically included phrases such as, “***thank God it is fine*, *though we now eat just once daily*; *thank God we’re coping fine though we withdrew some children from school***”. A 33year old participant explicitly stated this in her interview:

*“I thank God for the surgery though part of the money that we were supposed to use to pay school fees of the children*, *was among the money used for the payment of the bill”*. ***R30_F_NSD_33years_private***

In addition to normalization of adverse experiences, it was observed that consequences of catastrophic expenditure were attributed to fate or God. A participant said during an interview: “***Amu wa Olorun ni***” (meaning–“*whatever happens to an individual in life only happens with God’s knowledge and approval”*).

With regards to participants’ ideas on how experiences in the hospital might have been made easier, their responses were summarized under two main themes: “federal level policy or program reforms” and “Individual/Social level interventions”.

### Federal level policy or program reforms

*(i) National Health Insurance Scheme (NHIS)*. The current social health insurance scheme was believed by many of the participants to be operating sub-optimally. Interviewees described it as bureaucratic and inaccessible to many people. According to the participants, problems with NHIS were three-fold.

First, the issue of inadequate coverage; that NHIS is limited to public sector employees and the organised private sector only. This leaves too many households uncovered and with unpleasant experiences that could have been averted if freely available NHIS benefits were extended and available to everyone irrespective of class of work.

*“It is supposed to be available for everybody*, *not government workers alone*. *We are all Nigerians*, *so NHIS should be available for everybody—****R18_F_NSD_29years_public***

Secondly, for those that had coverage, it was unclear what medical/surgical issues were covered and which were not. Some participants paid for healthcare services and drugs themselves without knowing that the services were covered by the scheme.

*Even some of the drugs that we paid for*, *we were not supposed to because we are under NHIS but we ended up paying for them…… It was at end that we realized that we were not supposed to pay*…*……*. ***R12_F_NSD_28years_public***

Thirdly, participants were not certain of the mechanism of reimbursements, if they were to pay for services out of pocket and apply for refunds after discharge or if their assigned Health Management Organizations (HMO) were to take care of all medical expenses right from the period of admission. For those who were entitled to reimbursements after the services had been rendered, it was unclear how to process the refunds later.

*(ii) Government subsidy on health and access to soft loans*. Another option offered was the need for government to either subsidize the costs of emergency surgical care and or facilitate and the provision of soft loans to households undergoing emergency surgery. Three main suggestions were provided by participants:

Firstly, according to some of them, subsidization of hospital costs would be beneficial for families in need of financial assistance.

“*…I mean by drugs*, *money (subsidization of hospital costs)…do you understand*? *For instance you know a surgical cost of N150*, *000 that’s now subsidized to N80*, *000 will go a long way*.*…*.*”* ***R24_F_SD_37years_private***

“*What I am trying to explain is that for a federal hospital*, *it is a government hospital*, *so they should reduce the money*, *it is too much*. *We too we are managing*. *You can see the economic situation*, *how much are we earning that we are paying such huge amount of money*? *It is too much*, *they should reduce the cost or subsidize it”*.

***R20_F_NSD_29years_public***

Secondly, in addition to subsidization of hospital bills, participants also opined that the provision of soft loans through a dedicated fund scheme for families experiencing financial difficulty would suffice.

“*Organizations where access to quick or soft loans should be available for patients in such conditions”* ***“R23_M_SD_35years_private***

Thirdly, salary deductions that allow for flexible and spread out payments over a period of time after having required emergency care was another suggestion offered by the participants. According to the participants, steady monthly deductions from salaries will lessen the impact of hospital bills.

“*One can use some amount of money out of the monthly salary but they can also be deducting it from the person’s salary bit by bit”* ***R17_F_SD_46years_private***

*(iii) Upgrade of existing structures*. Some participants recommended that publicly funded and managed laboratories should be upgraded to provide subsidized laboratory services to the general public. In this way, not just the hospital bills will be subsidized but also the laboratory services that are also perceived to be expensive.

“*Let them work more on improving the laboratories so that we can pay for investigations in the lab and get it at the time we should get it because not everybody can afford the PPP lab*. *Even if I can afford it*, *of course*, *I want something cheaper and get quality”*.

- ***R15_F_NSD_32years_public***

#### Individual/Social level interventions

*Pre-informing*. Participants suggested that usual practice should change so that prior to hospitalization people should be informed about the cost and how hospitals will charge. They also wanted hospital bills to be standardized.

“*I think that during antenatal*, *they should tell people things to get in case of surgery*. *The same way they give list of things to buy for deliveries*, *they should also give list of things to buy for surgeries*. *So that we can know the cost of things we are going for”*.

***R12_F_NSD_28years_public***

*Appropriate billing*. In addition to recommending being pre-informed about bills, double and unsubstantiated bills were criticized by some participants:

“*Let them stop bringing out unsubstantiated bills*. *I got a bill that included things like my anesthesia*, *my IV fluids*, *things I bought myself*, *I feel cheated that I had to pay for those things when I have already provided them*, *it’s unfair…*.*”*.

***R15_F_NSD_32years_public***

## Discussion

Even though, slum dwellers are in general poorer than non-slum dwellers, we did not find differences in the experiences reported by slum and non-slum dwellers. This is perhaps explained by the fact that only those who experienced CHE were included in the study.

Our data indicated that prior to the advent of an emergency health care need, the idea of saving for a possible health-related need was unacceptable to participants. The notion of savings or any form of involvement in pre-payment schemes was not a predominant feature. Despite the fact that local savings clubs (such as “Esusu”, “Adashi”, “Otataje” or “Ajo” are popular within social networks [60], antipathy towards saving for a health related expenditure was observed. Local savings clubs essentially pool money through regular contributions and permit a one-time lump-sum collection at the end of a cycle [61]. However, these lump-sum payments are utilized preferably for capital projects such as purchase of land or erection of buildings rather than for health or health care purposes, again reinforcing that expenditure on health care is not prioritized. Also, these clubs operate within known social networks that differ from the idea of pre-payment into an anonymous fund, over which members feel they have no control. Although, evidence abounds in literature that the principle of solidarity found with mutual associations, cooperative societies [62] and microcredit organizations[63] have enormous potential to facilitate participation in health insurance schemes such as the community based health insurance schemes [64], our data does not corroborate this. As such, participation in initiatives such as the community based health insurance schemes continue to show low participation rates and ultimately the national solidarity programs like the NHIS [65].

After a health care need is established, the choice of a service was largely determined by word of mouth. Pertinent issues for participants were aesthetics and the human resource availability. None of our informants reported inquiring about costs when deciding which facility to seek care from. However, when we explored their suggestions to improve the system, cost issues were preeminent–recommending that adequate information on hospital costs and clear billing procedures should be made available upfront prior to commencement of care. Cost of health care has always been a very strong predictor of affordability of care. Contrary to findings from literature [7,66], pre-informed inquiries for cost of procedure was not sought or considered important to our study participants. The possibility for this variance could be due to the fact that our respondents were under some degree of pressure as their cases were surgical emergencies requiring urgent attention unlike the study participants in another study who were full time local government staff within a secretariat [7]. Urgency associated with surgical emergencies are usually associated with unusual dynamics that often influence decision making process or bargaining power among the end-users [67]. In a study conducted in East Midlands, patients scheduled for emergency surgery were significantly less likely to read or understand the consent form for surgery (nor felt they had a choice whether to sign or not), compared to their counterparts scheduled for elective surgeries [68].

Paying for services identified the central role of family and social support for many households in this study which confirms findings from the literature [69,70]. Though numerous studies have documented the positive influence of social support and social networks to relieve the payment of health care, evidence also exists that the high costs of care can negatively impact social networks. In a study conducted in Burkina Faso, it was seen that the challenge of meeting health care costs had led to the dissolution of relationships and breakdown of social links, leaving affected individuals in a state of heightened social and economic vulnerability [71].

The findings of this study revealed that there are three consequences of an inability to pay: delayed or poor quality care [72], humiliation of patients and incarceration. Delay in care by health workers to those unable to pay validates findings that has been described by others [72,73]. Humiliation as documented in this study has also been reported elsewhere in literature [74]. Inability to pay has been shown to prolong hospital stay (patients are not discharged but are incarcerated until they can pay). Surgical patients in rural Nigeria that experienced some difficulty with payments stayed in hospital seven days longer than those who could pay [75]. This phenomenon, is increasingly common [76] and has even resulted in the demand for sex in exchange for inability to pay for health care costs [77]. While this is accepted as a common practice that seems to result in little public protest, it is nonetheless increasingly being recognized as a human right violation [74].

Participants regardless of an ability to pay during admission complained about billing. Obscure billing procedures/discrepancies in the form of double billing and bureaucratic reimbursement systems were common experiences. Contrary to the widely accepted use of double billing in literature [78], (described as a process whereby a physician bills both the government and the patient for the same services), the use of the term, in this study, refers to billing the patient at least twice during the index admission for the same hospital services. Nonetheless, double billing in the context of this study, is not new and only serves to corroborate documented literature [79], which constitutes a type of fraud that undermines the trust of health care consumers and indicates lack of transparency of the system. With the exception of some kind of co-payment, that has a primary role to eliminate moral hazard [80], billing of patients could be eliminated if the existing social health insurance scheme was optimized.

Similar to other research, CHE that ensued, in this study, resulted in serious consequences such as going without food or taking children out of school [81]. The initial reaction when being questioned about unexpected and unaffordable charges was to appreciate being alive or to defer to the inevitability of the outcome. Participants normalized their experiences by attributing unforeseeable and unplanned circumstances to fate and thus rationalized key (positive and negative) life events–a common finding in this and other research [82,83].

The antipathy to save and willingness to pay for a health related expenditure only after it has occurred, as found in this study, contradicts the expressed desire to join the NHIS. The antipathy can be improved through federal level interventions and individual/social level interventions. At the federal level, there is a need for interventions that explain and encourage support for the principle of social solidarity (that is pre-payment, if care is sought or not, or whether any form of expenditure is incurred or not). Pooling of funds and social solidarity are essential preconditions for any sustainable health insurance system. If the people suggesting the extension of the existing NHIS as a solution are the same people who also resist pre-payment and health related savings, it suggests that they may not understand the principles underlying national health insurance schemes. This can limit uptake of NHIS and may explain why the prevalence of insurance cover is low in Nigeria.

At an individual level our findings revealed that “*soft loans*” (loans that can be paid back at one’s convenience rather than at predetermined dates and rates) are an acceptable intervention to alleviate health related expenditure. In any formal system, paying back when convenient is not tolerated which may render the concept of soft loans un-implementable. Short term or flexible loans are often associated with exorbitant interest rates which limits its’ use-value in offsetting CHE. The acceptability of a loan system to payback incurred expenditure again underlines that the notion of pooling of funds and cross subsidization is not understood or acceptable. It appears that the rudiments of health insurance are either poorly understood or participants are just not ready to “*walk the talk*”. Buy-in is required from health care consumers to gain and enjoy full benefits of the existing health insurance program. Proper education is required to improve scale up and coverage of the scheme [84]. Our data suggests that this would have been likely if high quality care was provided or if the scheme was more user-friendly with clear reimbursement systems. In order to achieve active voluntary participation and better coverage by the NHIS, those who are covered must be well served and protected from CHE.

### Limitations

The observed incidences where participants normalized their hospital experiences initially before probing may mean that they were influenced. Generally, interviewer bias is a bias that has repeatedly influenced such study designs [77]. It may also be the case that the participants only discussed the negative side of their experiences after probing because they initially did not trust the interviewers. There were also missed opportunities to probe further these themes. It may be argued that more themes might have emerged if probes were thorough or if other teams had conducted the interviews. Nonetheless, our study provided new insights on contextual factors that interact and influence the health-seeking choices, behaviours, and consequences of households facing emergency surgical care in sub-Saharan Africa.

## Conclusion

Evidence from this study establishes that catastrophic health expenditure does exist and causes similar effects to that which has been described in the literature. Catastrophic health expenditure occurs even for those with health insurance cover and this has the potential to undermine the perception and value of insurance. Patients have little to no knowledge of how much an emergency intervention will cost and there is no cost information available.

### Recommendations

In the short term the charges per day or per intervention should be made public at all points of service. In particular, during antenatal care, information about the cost and required preparation for a potential emergency intervention should be shared with service users. It is one of the few emergency surgical events that allows for preparation. Even in situations where people do not routinely save for health related expenses, this knowledge may prompt some kind of preparation.

Members of the various NHIS structures must be provided with correct information about what is covered, and how to claim reimbursements. It is equally important to understand why, in spite of insurance cover, some people still experienced CHE. Research on this is warranted and urgent, this may lead to changes in payment policies if required.

In the longer term if Nigeria is to surpass the approximately 4% membership of the NHIS, then efforts must be stepped up to ensure that the NHIS is well understood and administered and does not lead to CHE so that it is attractive to non-enrollees.

Also, more research should be done to confirm if the antipathy towards prepayment and health-related savings found in this study are common. If so, methods to encourage health related saving and pre-payment, have to be found. Simply assuming that prepayment, and social solidarity will be embraced because a policy exists seems unwise.

## Supporting information

S1 File Patterns of expenditure qualitative data. [85].
(DOCX)

## Abbreviations

*R15_F_NSD_32years_public*: Fifteenth participant; Female, Non-Slum dweller, 32years and sought care in a public secondary facility
*R24_F_SD_37years_private*: Twenty-fourth participant; Female, Slum dweller, 37years and sought care in a private secondary facility.
*R16_M_SD_27yrs_public*: Sixteenth participant; Male, Slum dweller, 27years and sought care in a public secondary facility
