# Supplementary material for: Affording unavoidable emergency surgical care – The lived experiences and payment coping strategies of households in Ibadan metropolis, Southwestern Nigeria
Source: PLoS One. 2020 May 20;15(5):e0232882. doi: 10.1371/journal.pone.0232882 (PMC7239385; doi:10.1371/journal.pone.0232882)
Supplement: S1 File — [85]. (DOCX) [file pone.0232882.s001.docx]

Patterns of Expenditure Qualitative Data. [85]

Available at [**https://doi.org/10.6084/m9.figshare.11417994.v1**](https://doi.org/10.6084/m9.figshare.11417994.v1)
